# Prevalence of HIV, Syphilis, HCV and Their High Risk Behaviors among Migrant Workers in Eastern China

**DOI:** 10.1371/journal.pone.0057258

**Published:** 2013-02-22

**Authors:** Xiaohong Pan, Yingying Zhu, Qing Wang, Hui Zheng, Xin Chen, Jing Su, Zhihang Peng, Rongbin Yu, Ning Wang

**Affiliations:** 1 Department of Epidemiology & Biostatistics, School of Public Health, Nanjing Medical University, Nanjing, Jiangsu, China; 2 Zhejiang Province Center for Disease Control and Prevention, Hangzhou, Zhejiang, China; 3 National Center for AIDS/STD Control and Prevention, Chinese Center for Disease Control and Prevention, Beijing, China; Public Health Agency of Barcelona, Spain

## Abstract

**Objective:**

The goal of this study was to understand the knowledge about AIDS, identify the correlates and determine the prevalence of HIV infection, syphilis, HCV among migrant workers in Zhejiang, China.

**Methods:**

A cross-sectional study using face-to-face anonymous questionnaire interviews was conducted and blood samples were collected for HIV, syphilis and Hepatitis C infection screening.

**Results:**

17,377 (92.8%) of 18,730 migrant workers approached were interviewed. Among 17,377 participants, the HIV/AIDS knowledge rate was 66.2%. A total of 12,694 (73%) of the participants reported having ever had sexual intercourse, with 30.1% of single participants reporting having had sexual intercourse. Among those respondents with sexual experiences, 7.5% admitted they had two or more sexual partners and 4.9% reported having had sex with casual (unpaid) partners in the previous 12 months, whilst 3.7% had paid for sex. More than half of those who had paid for sex (59.4%) had not used a condom every time in their sexual acts with the sex workers. Multiple logistic regression analysis indicated that high risk sexual behavior (defined as sex with a casual or commercial sex partner without using a condom consistently) was associated with being divorced or widowed (P<0.05 for single); male gender; shorter duration of stay in Zhejiang; working in factory, market or domestic service (P<0.05 for odd job); having a province of origin inside Zhejiang; and drug use. The prevalence of HIV and HCV infections were 0.02% (95% CI: 0.01%–0.06%) and 0.40% (95%CI: 0.31%–0.51%), respectively. The prevalence of syphilis among those who were sexually active was 0.55% (95% CI: 0.43%–0.70%). Risk factors for syphilis included shorter duration of stay in Zhejiang, ethnic minority status, being divorced or widowed and having had multiple sex partners.

**Conclusions:**

Much greater efforts are needed to promote safer sex, and programs for the control of syphilis need to be tailored for migrant workers in China.

## Introduction

Concurrent with the rise in international population mobility, over the past 30 years HIV has spread from high-risk nations to countries all over the world [1], [2], [3]. Furthermore, immigrants to a country and internal migrants can both spread the infection to the general indigenous population via concurrent sexual partners in their country of residence [4]. Internal migrants potentially facilitate HIV and sexually transmitted diseases (STDs) transmission between rural and urban populations through their annual return to their home villages [5], [6], [7]. Hence, migrants may serve as a "bridge" population transmitting HIV/STDs from high-risk groups to the general population.

The Chinese Ministry of Health (MOH) estimated that the number of people living with HIV (PLWH) increased from 650, 000 in 2005 to 780, 000 in 2011. The proportion of persons infected through sex is rapidly increasing as well, from 9.8% in 2005 to 81.6% in 2011 [8], [9]. Many social and economic elements in China facilitate the spread of HIV/AIDS through sexual transmission. These elements can include the prevalence of STDs [10], [11] and the rate of population mobility [12], [13]. High mobility among rural-to-urban migrants was thought to increase high-risk sexual activities in previous studies [14], [15], [16]. It is estimated that there are approximately 220 million migrants in China [17]. Knowledge about the HIV/STDs epidemic status is urgently needed to develop an appropriate prevention and control program, as well as to provide valuable evidence for allocating resources for AIDS treatment and behavioral interventions among migrants. Although previous surveys in China suggested that a large number of migrants were willing to be tested for HIV/STDs [18], [19], and 4,293 VCT(voluntary counseling and testing) clinics had been established throughout the country by the end of 2007 [20], migrants still have less access to HIV/STD tests and treatment due to their low awareness of VCT services and their subjection to a household registration system in which migrant workers are not entitled to social security or medical benefits [20]–[21]. Most previous epidemiologic and behavioral surveys of HIV/STDs in China have focused on migrant MSM, migrant female sex workers or migrant workers working in a certain occupation with a small sample size, but were rarely based on a large samples that included almost all subgroups of migrants in eastern China where there is a large assemblage of this group [22], [23], [24].

Therefore, the aim of this study is to fill the gap on the prevalence of HIV/STDs and related risk factors among migrant workers in economically developed eastern China.

## Methods

### Study Site and Sample

Zhejiang is a coastal province that experienced early rapid economic growth and is a magnet for migrant populations. In 2009, there were over 20.06 million migrants who moved from rural to urban areas of Zhejiang for jobs [17], [25]. The total number of migrants in Zhejiang has been the second largest in China since 2001 [26].

Migrant workers in this study were defined as people born and registered as permanent residents in a rural area, but who had currently been working in legal jobs in the county seat of Zhejiang for at least three months. After meeting with and obtaining agreements from local government officers, venue managers, and administrators, a preliminary study was conducted among migrant workers to better understand their socio-demographic characteristics and working and living situations, which would be used for selecting a study sample. The preliminary study and migrant management revealed that migrant workers in Zhejiang were primarily engaged in six broad venue categories: construction industry (21%), mining (0.2%), factory (35%), market (0.4%), domestic service (20%) and miscellaneous jobs (18%). These six occupational clusters accounted for more than 90% of migrants in Zhejiang and were selected as the main sampling frame. The formal study was carried out in 33 counties or districts of the province’s 11 prefecture-level cities where their samples were representative. Quota sampling was employed for original sample selection in these areas. For construction industry, mining, factory and domestic service, the research teams in each area used workplaces (construction site, mining, factory, store, restaurant, or club) as the sampling units. Marketers who had applied for and obtained the business licenses were recruited from the industrial and commercial administrations. Migrant workers’ markets were used as the sampling units for miscellaneous jobs. At the beginning of our study recruitment, there were 100 construction sites, 11 mining, 150 factory, 11 prefecture-level industrial and commercial administrations for selecting samples of marketers, 99 workplace for selecting samples of domestic services and 33 migrant workers’ markets for selecting samples of miscellaneous workers asked to participate and none refused. To reduce the sampling bias, it was required that the number of participants recruited in each occupational cluster in the sample be proportional to the overall estimated distribution in the entire mobile population. The numbers of migrants recruited from any unit did not exceed 20% of total migrants in the unit. Interviewers provided eligible individuals with a detailed description of the study design and invited them to participate. A total of 18,730 migrant workers from the above six broad venue categories were approached and 17,986 of them voluntarily joined this study and provided blood samples. The number of participants recruited in each occupational cluster was considered proportional to the overall estimated distribution in the entire mobile population. Thus, we finally identified 17377 of 18730migrant workers who participated in the study. Of the participants4035 (23.2%) were engaged in construction sites, 42 (0.2%) in mining, 6568(37.8%) in factory, 72 (0.4%) in market, 3538 (20.4%) in domestic service, 3122 (18%) in miscellaneous jobs.

### Ethics

This study was reviewed and approved by the ethics committee of Zhejiang Bureau of Public Health and Zhejiang University. The objectives and procedure of the study and the potential risk and benefits of participation were given to potential participants during recruitment of study subjects samples. Written informed consents were obtained from all subjects participating in the survey.

### Data collection

The main study was administrated from March to June 2010. Respondents were asked first to complete a questionnaire, which was anonymous and composed of four parts. Parts one and two were designed to obtain information about socio-demographic characteristics and knowledge of HIV/AIDS. Part three was about intra-marital and extramarital sexual behaviors and condom use, including casual sex, commercial sex and homosexual sex. Part four was about drug use. Participants chose either a self-administered interview or a face-to-face interview in private rooms at their workplace or at a nearby place convenient to them. The interviewers were public health workers who had experience with administering epidemiological questionnaires. The completed questionnaires were deposited into a large closed box with other responses, demonstrating to the participant that responses could not be viewed by others.

Before blood samples were taken, all participants were provided with information about HIV/STDs, and an information leaflet was provided. All were offered freepost-test consultations and informed that they could receive their test results and free treatment if found to be positive. All participants needed to leave their telephone numbers and passwords to receive their test results. The password was serviceable in proving the identity of the participant and protecting their privacy. Testing for HIV, syphilis and HCV was offered on an anonymous basis. Consent was then obtained and venous blood was collected by professional healthcare workers using disposable sterile needles and tubes for testing HIV/AIDS, syphilis and HCV. Among those who tested positive for AIDS and syphilis, only three syphilis patients could not be offered free treatment because they changed their phone numbers.

### Laboratory Testing

Blood samples were screened for HIV and HCV antibodies using an enzyme-linked immunosorbent assay technique (ELISA; bioMerieux, The Netherlands) according to the manufacturer's instructions. Any samples that screened positive for HIV and HCV were confirmed by a Western blot assay (HIV BLOT 2.2; Genelabs Diagnostics Pte Ltd., Singapore)and an enzyme-linked immunosorbent assay technique (ELISA; Zhuhai Livzon, Zhuhai, China), respectively. Syphilis screening was performed by rapid plasma reagin (RPR; Shanghai Rongsheng, Shanghai, China) and confirmed by Treponemapallidum particle assay test (TPPA; Fujirebioinc, Japan) [27]. All tests were performed in the HIV/STD Laboratory of Zhejiang Provincial Center for Disease Control and Prevention.

### Data analysis

HIV/AIDS knowledge was measured by eight questions ( they were about the three transmission modes of HIV, two on prevention activities for HIV through using condoms and keeping a regular sexual partner, two regarding misconceptions about HIV transmission through mosquito bites and eating with an HIV-infected individual, and one on HIV diagnoses and whether a person could be diagnosed as having HIV only from visual observation). The total score for HIV knowledge ranged from 0 to 8 (a score of 0 was assigned for a wrong answer or an answer of “unknown” or “unsure,” and 1 for a correct answer). These items were adapted from the scales used in China’s National HIV Surveillance Surveys [28].

### Statistical analysis

Descriptive analyses were conducted to elucidate socio-demographic characteristics and sexual risk behaviors for HIV. Bivariate analyses (Chi-squared test or Fisher’s exact test, depending on which was more appropriate) and logistic regression analysis were performed to estimate the crude odds ratios (cORs), adjusted ORs (aORs), and 95%confidence intervals (CIs) of potential risk factors associated with high risk sexual behavior among migrant workers, syphilis and HCV infections, respectively. All statistical analyses were carried out using the SPSS system for Windows Version 17.0. The significance level was defined as *P* = 0.05.

## Results

### Description of study participants

Table 1 presents and compares socio-demographic characteristics by gender. The sample consisted of 63.3% males and 36.7% females, with a mean age of 31.04 years (SD = 10.07), and 75.4% were less than 38 years of age. Most participants were of Han ethnicity (95.3%) and had received a junior high school education or below (76.2%). About 30.7% were single and 61.4% had lived in Zhejiang longer than one year. The proportions of study participants who came from Anhui and Henan provinces, which have both been identified as provinces of infected former paid plasma donors, were respectively 17.2% and 11.8%. Paid plasma donation was previously a major mode of HIV transmission in China. The proportions of study participants falling into each venue category were approximately 37.8% for factory; 23.2% for construction industry; 20.4% for domestic service; 18.0% for miscellaneous job; 0.4% for market and 0.2% for mining. Compared to male migrants, female migrants in the study were more likely to be employed as miscellaneous-job workers (*χ^2^* = 33.130, P<0.001). Males and females were significantly different in all social and demographic characteristics excluding province of origin.

**Table 1:** Socio-demographic characteristics and HIV/AIDS-related knowledge by gender.

	Male(N = 11006)	Female(N = 6371)	P
	No.(%)	No.(%)	
Age (years)			<0.001
∼18	555(5.0)	470(7.4)	
19∼29	4598(41.8)	3186(50.0)	
30∼39	3186(28.9)	1569(24.6)	
40∼	2677(24.2)	1136(18.0)	
Marital status			<0.001
Single	3241(29.4)	2088(32.8)	
Married	7217(65.6)	3957(62.1)	
Cohabiting	507(4.6)	308(4.8)	
Divorced or widowed	41(0.4)	18(0.3)	
Duration of stay in Zhejiang (yrs.)			<0.001
∼1	4544(41.3)	2169(34.0)	
1∼	6462(58.7)	4202(66.0)	
Ethnicity			0.015
Minority	545(5.0)	264(4.1)	
Han	10461(95.0)	6107(95.9)	
Education			<0.001
Illiterate	309(2.8)	292(4.6)	
Primary school	1731(15.7)	1221(19.2)	
Junior high school	6294(57.2)	3381(53.1)	
Senior high school	2189(19.9)	1170(18.4)	
Above senior high school	483(4.4)	307(4.8)	
Migrants’ provinces of origin			0.147
Anhui	1841(16.7)	1147(18.0)	
Henan	1284(11.7)	768(12.1)	
Zhejiang	1142(10.4)	655(10.3)	
Sichuan	1125(10.2)	609(9.6)	
Other provinces	5614(51.0)	3192(50.1)	
Occupation			<0.001
Construction worker	3368(30.6)	667(10.5)	
Miner	18(0.2)	24(0.4)	
Factory	4157(37.8)	2411(37.8)	
Market	64(0.6)	8(0.1)	
Domestic service	1562(14.2)	1976(31.0)	
Miscellaneous job	1837(16.7)	1285(20.2)	
HIV/AIDS knowledge (score)			<0.001
0–5	3480(31.6)	2399(37.7)	
6–8	7526(68.4)	3972(62.3)	

### Knowledge about HIV/AIDS

Most (70.4%) of the participants knew that an HIV-infected individual could not be diagnosed through visual appearance. Approximately 79.0% of them knew that HIV could be transmitted from a pregnant woman to her child and 86.5% knew it could be transmitted by sharing a syringe with an HIV-infected individual. Approximately 86.0% of them understood that HIV could be transmitted through blood transfusion, but only 65.3% recognized HIV could not be transmitted by sharing food. The question about whether HIV could be transmitted through mosquito bites was usually answered incorrectly by study participants;about53.8% thought that it was a mode of transmission. More than three quarters of migrants (76.2%) thought HIV could be prevented by using condoms consistently and 73.1% thought it could be prevented by keeping a regular sexual partner. In general, females had less knowledge about HIV/AIDS than males (Table 1).

### HIV-related risk behaviors

This section asked about practices that potentially pose direct risks for HIV transmission, as well as about behaviors that may lead to direct risk-taking.

A total of thirty-three respondents (0.2%) had ever taken drugs, while only eight (24.2%) admitted having ever injected drugs and seven of those had shared a syringe or needle. Approximately 71.4% of those who had injected drugs reported they had shared a syringe at some time in the previous 6 months, and 28.6% had never shared syringes. In addition, the prevalence of drug use was higher in both male clients of FSWs (0.9 vs. 0.2, *P*<0.01) and those who had ever had a casual partner (1.3 vs. 0.2, *P*<0.01).

Most (12694/17377) of the participants reported having ever had sexual intercourse, and amongst them30.1% were unmarried .Among those respondents with sexual experience, 11,727 (92.4%) reported having had only one sexual partner in their lifetime, and 91% (11552/12694) reported sexual intercourse with their wife or live-in partner during the past year. Of those with sexual experience, 616 (4.9%) reported having had sex with 'nonregular' (unpaid) partners during the previous 12 months. No women admitted a history of commercial sex with males. Of the 8,331 sexually experienced male migrants, 470 (5.7%) reported having had sex with a (paid) sex worker (SW) at least once during the previous 12 months, while 31 (0.4%) male participants admitted to sex with males in the last year. Of these 31 male migrants, 41.9% had bought sex in the last year. Unmarried participants were more likely to have had casual or commercial sex in the past year than currently or ever-married participants (Table 2).

**Table 2:** Sexual behavior and condom usage by marital status.

	Unmarried	married	P
	No.(%)	No.(%)	
Ever had sexual intercourse (n = 12694)			<0.001
Yes	1848(30.1)	10846(96.6)	
No	4296(69.9)	387(3.4)	
Condoms use with their spouse or live-in partner in the last year (n = 11552)			<0.001
Always	510(30.3)	5369(54.4)	
Sometimes	922(54.8)	3889(39.4)	
Never	250(14.9)	612(6.2)	
Among those with sexual experience, condom use at last sexual intercourse (n = 12694)			<0.001
Yes	773(41.8)	2454(22.6)	
No	1075(58.2)	8392(77.4)	
Ever had casual partners (n = 12694)			
Yes	228(12.3)	388(3.6)	
No	1620(87.7)	10458(96.4)	
Had unsafe casual sex in the last year (n = 12694)			<0.001
Yes	170(9.2)	286(2.6)	
No	1678(90.8)	10560(97.4)	
Ever had commercial sex (n = 8331)			<0.001
Yes	99(7.8)	371(5.3)	
No	1172(92.2)	6690(94.7)	
Had unsafe commercial sex in the last year (n = 8331)			0.002
Yes	61(4.8)	218(3.1)	
No	1209(95.2)	6843(96.9)	

Unsafe casual or commercial sex was defined as a participant with sexual experience having had casual or commercial sexual intercourse during the previous 12 months without using a condom every time.

Among the 11,552 sexually active respondents, 51.0% had never used condoms during intercourse with a regular sexual partner (wife or live-in partner) in the past year, and only 7.4% had always used them. Of the 616 with 'nonregular' (unpaid) partners, 25.2% had never used a condom in the previous 12 months and only 25.9% had used a condom every time. Results are similar for the 31 male participants who had had sex with males; only 19.4% had always used condoms. The level of condom use among those with a history of commercial sex in the previous 12 months was comparatively higher; 40.6% had used a condom every time in the last 12 months, whilst 59.4% had not.

During the most recent sexual intercourse, only 25.4% of the participants always used condoms with a regular partner, whereas 74.6% did not. Condoms were used by approximately half (57.6%) of those migrants with unpaid sexual partners at the most recent sexual intercourse. At the most recent commercial sexual encounter, 36.6% of them did not use condoms. Also, 41.9% of male respondents who had ever had sex with another male did not use condoms with a man at the most recent sexual encounter.

### Prevalence of HIV, syphilis and HCV

Among the 17,377 study participants, the prevalence of HIV and HCV were 0.02% (95% CI: 0.001%-0.006%) and 0.4% (95%CI: 0.31%-0.51%), respectively. The prevalence of syphilis among those who were sexually active was 0.55% (95% CI: 0.43%-0.70%). Migrants who had a history of sex with casual partners had significantly higher prevalence than those who did not of both positive HCV samples (1.8 versus 0.4, P<0.01) and positive syphilis clinic samples (1.9 versus 0.5, P<0.01). Males who had engaged in commercial sex behaviors also had a higher prevalence of syphilis (3.4 versus 0.4, P<0.01) and HCV (1.3 versus 0.4, P<0.01).

### Multivariate analyses

High risk sexual behavior among migrant workers was defined as a participant having had commercial or casual sexual intercourse during the previous 12 months without using a condom consistently. Socio-demographic characteristics, knowledge about HIV/AIDS and drug use appeared as variables in this logistic regression analysis. The result of the logistic regression analysis is presented in Table 3. Migrant workers with sexual experience who were divorced or widowed (P<0.05 for single migrant workers), male, had a shorter duration of stay in Zhejiang, were working in factory, market or domestic service positions (P<0.05 for odd-job workers), had a province of origin inside Zhejiang and had ever taken drugs were more likely to have had high risk sexual behaviors in the last year (Table 3).

**Table 3:** Risk factors for high risk sexual behaviors.

	N	High risk sexual behaviors	OR (95%CI)**	AOR(95%CI)***	P
		No.(%)			
Sex					
Female	4363	128(2.9)	1	1	
Male	8331	543(6.5)	2.307(1.896∼2.807)	2.409(1.960∼2.961)	<0.001
Age					
40∼	3669	166(4.5)	1	1	
30∼39	4530	187(4.1)	0.909(0.734∼1.125)	0.936(0.750∼1.168)	0.556
19∼29	4391	303(6.9)	1.564(1.288∼1.900)	1.122(0.877∼1.435)	0.360
∼18	104	15(14.4)	3.557(2.014∼6.281)	1.312(0.696∼2.471)	0.401
Marital status					
Single	1047	163(15.6)	1	1	
Married	10795	448(4.2)	0.235(0.194∼0.285)	0.286(0.225∼0.364)	<0.001
Cohabiting	801	48(6.0)	0.346(0.247∼0.484)	0.351(0.249∼0.495)	<0.001
Divorced or widowed	51	12(23.5)	1.669(0.855∼3.255)	2.039(1.010∼4.117)	0.047
Duration of stay in Zhejiang (yrs.)					
∼1	4225	270(6.3)	1	1	
1∼	8439	401(4.8)	0.736(0.628∼0.863)	0.845(0.717∼0.995)	0.043
Education (yrs.)					
≤6	3195	147(4.6)	1	1	
7∼9	7005	354(5.1)	1.104(0.906∼1.344)	0.818(0.660∼1.014)	0.067
>9	2494	170(6.8)	1.517(1.208∼1.904)	0.859(0.662∼1.116)	0.255
Occupation					
Miscellaneous job	2210	101(4.6)	1	1	
Domestic service	1933	136(7.0)	1.580(1.212∼2.060)	1.482(1.120∼1.960)	0.006
Market	60	8(13.3)	3.212(1.486∼6.943)	2.856(1.298∼6.284)	0.009
Factory	5020	281(5.6)	1.238(0.981∼1.563)	1.413(1.110∼1.800)	0.005
Miner	32	0(0.0)	0.000(0.000∼)	0.000(0.000∼)	0.998
Construction worker	3439	145(4.2)	0.919(0.709∼1.192)	0.905(0.689∼1.189)	0.474
Migrants’ provinces of origin					
Not in Zhejiang	11328	577(5.1)	1	1	
Zhejiang	1366	94(6.9)	1.377(1.099∼1.725)	1.288(1.016∼1.632)	0.037
Drug use					
No	12665	664(5.2)	1	1	
Yes	29	7(24.1)	5.751(2.448∼13.510)	3.758(1.501∼9.409)	0.005

High risk sexual behavior was defined as sex with a casual or commercial sex partner without using condom consistently

* The variables of *P*-value ≤0.1 were listed in this table by the univariate analysis. *P*-values ≤0.05 were considered statistically significant finally by multiple logistic regression analysis.

** Odds ratio was derived from the univariate analysis without controlling for potential confounders.

*** Odds ratio was adjusted for other variables listed in this table by multiple logistic regression analysis.

CI  =  confidence interval.

A logistic regression model was used to analyze factors related to syphilis infection among those who had sexual experience. Knowledge of HIV/AIDS and certain sexual behaviors were considered as intermediate variables in this specific logistic regression analysis. The result of the logistic regression analysis is presented in Table 4. The prevalence of syphilis was higher among participants with shorter duration of stay in Zhejiang, ethnic minority status and those who were divorced or widowed.

**Table 4:** Logistic regression analysis of syphilis.

	N	Syphilis Cases/Tested	OR(95% CI)**	A OR(95% CI)***	P
Age (yrs.)					
∼29	4495	32(0.7)	1	1	
30∼	8199	38(0.5)	0.649(0.405∼1.041)	0.867(0.487∼1.542)	0.627
Marital status					
Single	1047	15(1.4)	1	1	
Married	10795	47(0.4)	0.301(0.168∼0.540)	0.369(0.186∼0.735)	0.005
Cohabiting	801	4(0.5)	0.345(0.114∼1.044)	0.354(0.117∼1.072)	0.066
Divorced or widowed	51	4(7.8)	5.855(1.871∼18.327)	8.213(2.377∼28.377)	0.001
Duration of stay in Zhejiang(yrs.)					
∼1	4255	37(0.9)	1	1	
1∼	8439	33(0.4)	0.448(0.280∼0.717)	0.480(0.298∼0.772)	0.002
Ethnicity					
Minority	611	9(1.5)	1	1	
Han	12083	61(0.5)	0.339(0.168∼0.687)	0.373(0.183∼0.763)	0.007

* The variables of *P*-value ≤0.1 were listed in this table by the univariate analysis. *P*-values ≤0.05 were considered statistically significant finally by multiple logistic regression analysis

** Odds ratio derived from the univariate analysis without controlling for potential confounders.

*** Odds ratio was adjusted for other variables listed in this table as potential confounders.

CI = confidence interval.

Table 5 displays the results from the logistic regression models. In the model for HCV infections, socio-demographic characteristics, extramarital sexual behaviors and drug use behaviors were significant predictors for HCV infection among study participants. The result of the logistic regression analysis indicates that the prevalence of HCV was higher among participants who had extramarital sexual behaviors or had taken drugs.

**Table 5:** Logistic regression analysis of HCV infection.

	OR(95% CI)**	A OR(95% CI)***	P
Extramarital sex			
Yes vs. no	4.338 (2.406∼7.821)	4.045 (2.221∼7.365)	<0.001
Drug use			
Yes vs. no	16.391 (3.846∼69.849)	10.256 (2.309∼45.549)	0.002

* Statistically significant at α = 0.05.

** Odds ratio derived from the univariate analysis without controlling for potential confounders.

*** Odds ratio adjusted for socio-demographic characteristics as well as exmarital sexual behaviors and drug taking as potential confounders.

CI = confidence interval.

## Discussion

As an economically developed province and a magnet for migrant workers in eastern China, Zhejiang has recently been experiencing a more and more severe HIV/AIDS epidemic. Officially reported new HIV/AIDS cases have been increasing at an average annual rate of 30% to 40% in three years, rising to 8727 cases in 2011; however, the real number is probably much higher. By the end of 2011, experts estimated that 14,000 people are living with HIV/AIDS (PLHIV) in Zhejiang. Since migrant workers were seen as another vulnerable group for HIV related risky sexual behavior in addition to FSW and MSM groups [29], [30], this study provided more comprehensive data about the HIV/AIDS epidemic and the behavioral status among migrant workers group in Zhejiang.

In this study, 4.9% of participants with sexual experience reported casual sex in the past year, whilst 3.7% had paid for sex. However, only 25.9% and 40.6% used condoms consistently in the above two sexual activities, respectively. Another survey of 4187 migrant workers in Beijing showed a similar result, in which 4.3% engaged in unpaid casual sex and 5.6% had commercial sex in the past year [31]. The non-optimistic behavioral status indicated a future risk for HIV/STDs infection among migrant workers in Zhejiang, thus giving a preliminary suggestion of a need for behavioral interventions in those migrant workers who had high risk sexual behaviors.

In this study, high risk sexual behavior was defined as sex with a casual or commercial sex partner without using condom consistently. Consistent condom usage has been recognized as an effective way to prevent the transmission of HIV/STDs, so it is essential to consider condom usage when identifying high risk sexual behaviors among migrant workers This is the first report encompassing condom usage when studying high risk sexual behaviors among migrant workers. From previous studies’ data and official figures, a large proportion of married migrants are generally accompanied by a spouse if they stay in one place for longer periods of time [32], [33]. The apparently high rate of high risk sex among participants who lived less than a year in Zhejiang supports this theory. The mixed-gender character of this population not only reduced extramarital sexual encounters, but also may alter the sexual culture. This behavior is underpinned by the fact that single, divorced or widowed migrant workers are more likely to seek out sex with casual or commercial sexual partners [30], [34]. Unfortunately, the exposure rate of high risk sex was relatively high among these single and divorced or widowed participants in our survey. Our findings suggest that participants who had a province of origin inside Zhejiang were more susceptible to having high risk sex intercourse. There are two explanations: 1) Zhejiang is an economically developed province in China: its GDP per-capita of $6,501 is far higher than other provinces [35] and these migrants have a better economic status than those coming from other provinces and therefore have more opportunities to purchase sex; or 2) Local rural residents have more extensive interpersonal relationship networks which may facilitate casual sex. Also, they are more familiar with brothels and other sites of commercial sexual activity than the migrants from other provinces. Moreover, we found that a migrant with a stable job or reported history of drug abuse was more likely to have risky sex.

All of these findings have important implications to develop pragmatic measures for HIV/STD prevention and control among migrant workers in Zhejiang and other areas. Firstly in general, the control of HIV/STDs among migrant workers should be conducted through a coordinated effort between the government, health department, and community as well as employers. In particular, the community should engage in the organization, management and implementation of such work, while health professionals provide technical support and employers give site support and some other convenient support efforts. Furthermore, the government should foster a calibrated combination of rewards and punishments to ensure this work goes effectively. Secondly, targeting subgroups prone to high risk sex such as single and divorced or widowed migrants, free condom distribution should go hand-in-hand with health education materials on the importance of maintaining single sexual partners and using condoms consistently with both casual and commercial sexual partners. Of course primary sex education such as how to use a condom correctly should also be added to this process, considering that the absence of general sex education for young people is an indisputable fact in China. Thirdly, policies should encourage employers to provide affordable housing for newly arrived married employees in order to facilitate healthy sexual relations as part of a balanced home life. Finally, we also should pay attention to drug user subgroups among migrant workers, and provide economic support and psychological care to assist them with detoxification.

On the serological survey, the prevalence of HIV infection among migrant workers in Zhejiang (0.02%)was far below that of India (2.20%), Nepal (2.80%), and Thailand (4.90%) [4], [36], [37]. The disparity may be due to different sample characteristics, different traditional cultural backgrounds, and different socioeconomic environments. The prevalence of HIV infection in this study was a little higher than the reported rate in the general population in Zhejiang (0.01%). In addition, the prevalence of syphilis among migrant workers who were sexually active was 0.55%, higher than the estimated 0.02% among the general population of Zhejiang. Syphilis is regarded as an indicator of unprotected sex and the potential for HIV to spread within a population, so there is a risk of further increasing the prevalence of HIV/AIDS in this group [38]. A study in Beijing revealed that stable employment and female gender were associated with syphilis among migrants [39]. Our survey showed that syphilis was significantly associated with shorter duration of stay in the study site and divorced or widowed marital status, which is consistent with the fact that these two subgroups were more prone to high-risk sex in our findings. Such disparities between different studies reflect the complexity of correlations between risk factors and STD infections. In addition, the prevalence of syphilis was higher among migrants with ethnic minority status, which was similar to studies of other countries [40], [41], [42], [43]. Therefore, STI prevention and control programs need to be strengthened among these migrants.

Hepatitis C virus is a major health problem worldwide, particularly in Asian countries such as China [44]. Plasma donation and sexual transmission were major modes of HCV transmission in China. Based on previous studies, we realized that for migrants mainly from those provinces where infected former plasma donors (FPD) have been identified [45], understanding the prevalence of HCV in the migrant population is exigent. To the extent of our knowledge, this is the first report of a community-based study of HCV prevalence among migrants working in various vocations in China. Our study demonstrated that HCV infection among rural to urban migrants was significantly associated with having extramarital sexual behaviors and having taken drugs. The prevalence of HCV among migrants was 0.40% (95%CI: 0.31%-0.51%). Nevertheless, it is reported that the overall estimated prevalence rate of HCV in China is 3.20% [46]. At the root of the issue may be the self-selection of migrants that affects health in two ways. First, young and healthy people are more likely to migrate than elderly people or people with disabilities or chronic illnesses. Second, more serious and incapacitating diseases and intensive-care conditions could result in a migrant returning home to their village to seek family support and avoid the high medical and living costs in cities [47], [48].

The study has several limitations. Firstly, although the study achieved a large sample size, we did not use a random sampling method to select the study population, which might have led to selection bias. Secondly, accurate information about sexual behavior can be very difficult to obtain despite guaranteed anonymity, leading to a possible reporting bias. Finally, the cross-sectional nature of the study made it difficult to determine a causal relationship. In spite of these limitations, however, our findings have important public health implications for HIV/STD intervention programs.
